# Light Chain Escape in 3 Cases: Evidence of Intraclonal Heterogeneity in Multiple Myeloma from a Single Institution in Poland

**DOI:** 10.1155/2015/809840

**Published:** 2015-12-31

**Authors:** Maria Kraj, Barbara Kruk, Kelly Endean, Krzysztof Warzocha, Katarzyna Budziszewska, Monika Dąbrowska

**Affiliations:** ^1^Institute of Hematology and Transfusion Medicine, Ulica Indiry Gandhi 14, 02-776 Warsaw, Poland; ^2^The Binding Site Group Ltd., 8 Calthorpe Road, Birmingham B151QT, UK

## Abstract

We report three cases of light chain escape (LCE) at a single institution in Poland, including an interesting case of biclonal monoclonal gammopathy of undetermined significance (MGUS) that satisfied the criteria for progression to light chain multiple myeloma (LCMM) with a rapid rise in serum free light chain (FLC) levels, following steroidal treatment for simultaneous temporal artery inflammation and polymyalgia rheumatica (PMR). In the three cases discussed, progression of the disease by light chain escape was associated with rapid and severe renal impairment, highlighting the necessity for prompt detection of such free light chain-only producing clones in order to prevent the possible development of irreversible end-organ damage. Interestingly, monitoring of these three patients by serum free light chain assay (sFLC) and retrospective heavy/light chain analysis (HLC) detected this clonal evolution prior to clinical relapse and suggests that these assays represent important additional tools for more accurate monitoring of multiple myeloma patients.

## 1. Introduction

Multiple myeloma (MM) is characterised by the production of a monoclonal protein which could be an intact immunoglobulin, free light chain (FLC), both, or neither. MM is almost always preceded by a premalignant disease called monoclonal gammopathy of undetermined significance (MGUS), an incidental laboratory finding also characterised by the production of a monoclonal protein [1, 2]. MGUS may evolve to MM or other B cell lymphoproliferative diseases and this evolution is thought to be due to the acquisition of genetic mutations by the tumour cell clones and associated changes in the bone marrow microenvironment [3, 4]. Originally this transformation from MGUS to MM was considered to occur in a linear fashion; however emerging evidence suggests that disease evolution follows a Darwinian-like branching process giving rise to multiple clones present at MM diagnosis [5, 6]. A study by Ayliffe et al. identified the presence of dual populations in the bone marrow of a proportion of MM patients, where bone marrow plasma cells (BMPCs) produced either monoclonal FLCs or intact immunoglobulins, indicating the presence of separate clones [7]. The discovery of this intraclonal heterogeneity in MM suggests that serological analysis could act as a surrogate marker for bone marrow tumour cell populations [7, 8].

Intraclonal heterogeneity may impact response to treatment, including disease progression and relapse, as the independent clones may have different response kinetics to treatment resulting in changes in clonal dominance [9]. Such clonal change was first described by Hobbs in 1971 during the first MRC myeloma trial. He reported that 5% of intact immunoglobulin MM patients relapsed with only Bence Jones proteinuria. It was termed Bence Jones escape and is now more commonly referred to as light chain escape (LCE) [10–12], defined as an increase in monoclonal FLCs without a corresponding increase in monoclonal intact immunoglobulins. In the most recent MRC myeloma trial, the incidence of LCE was 6.5% for IgG and 19.9% for IgA [10].

Here we report three cases of LCE observed at a single institution in Poland: 2 MM patients and a rare biclonal MGUS. These cases serve to highlight the importance of utilising sensitive monitoring tools capable of detecting clonal change at an early stage in order to allow therapeutic intervention aimed at preventing irreversible end-organ damage.

## 2. Case Presentation

### 2.1. Case 1

**Figure 1:**
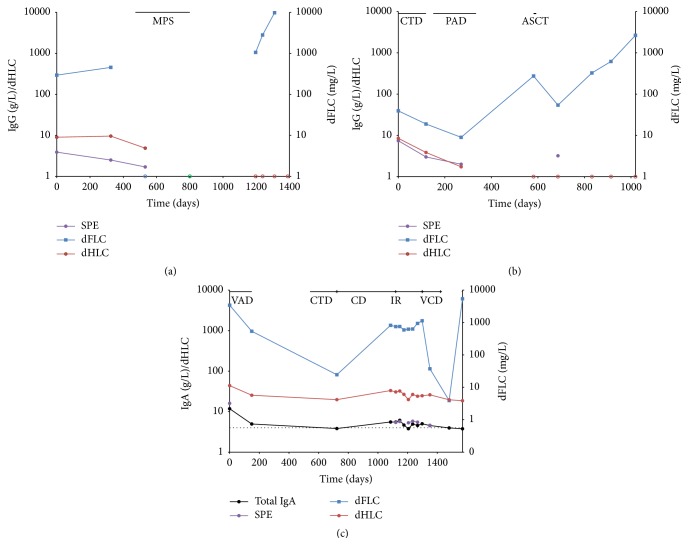
Disease course of patients with (a) biclonal MGUS progressing to LCMM (case 1), (b) oligosecretory IgG*κ* MM (case 2), and (c) IgA*κ* IIMM (case 3). MGUS: monoclonal gammopathy of undetermined significance, LCMM: light chain multiple myeloma, MM: multiple myeloma, IIMM: intact immunoglobulin multiple myeloma, SPE: serum protein electrophoresis, dFLC: difference in concentration between involved and uninvolved free light chain measurement (Freelite), dHLC: difference in concentration between involved and uninvolved heavy/light chain measurement (Hevylite), MPS: methylprednisolone, CTD: cyclophosphamide, thalidomide, and dexamethasone, PAD: bortezomib, doxorubicin, and dexamethasone, VAD: vincristine, doxorubicin, and dexamethasone, CD: cyclophosphamide and dexamethasone, IR: ionising radiation, VCD: bortezomib, cyclophosphamide, and dexamethasone, and ASCT: autologous stem cell transplant; blue open circle indicates a normalised sFLC ratio, red open circle indicates a normalised HLC ratio, and green open circle indicates normalised sFLC and HLC ratio.

A 71-year-old woman was hospitalised with a diagnosis of hypertension and coronary artery disease; routine haematological investigations identified an IgG*κ* monoclonal protein (SPE: 3.9 g/L) and monoclonal *λ* sFLC (*λ* sFLC concentration 316 mg/L; *κ*/*λ* sFLC ratio 0.07). Retrospective HLC analysis identified an abnormal HLC ratio (IgG*κ*/IgG*λ* HLC ratio; 4.75). A bone marrow biopsy revealed a 3% monoclonal plasma cell infiltration; a bone survey was negative for osteolysis and haemoglobin, calcium and creatinine levels were all normal. The patient was diagnosed with a biclonal MGUS (low/moderate risk) and was followed up annually by SPE, in accordance with IMWG guidelines (Figure 1(a) and Table 1). A year following diagnosis, the IgG*κ* monoclonal protein concentration was stable but, by contrast, the dFLC (involved FLC-uninvolved FLC) concentration had increased to 452.9 mg/L. Five months later, the patient was diagnosed with temporal artery inflammation and polymyalgia rheumatica (PMR) and treated with oral methylprednisolone for 11 months. The steroid treatment resolved the PMR and, coincidently, caused a reduction in the IgG*κ* concentration (trace quantities detectable by IFE) and normalisation of the *κ*/*λ* sFLC ratio (0.55) and IgG*κ*/IgG*λ* HLC ratio (1.34). A year after steroidal treatment, whilst the IgG*κ* monoclonal protein concentration remained stable (trace by IFE, normal IgG*κ*/IgG*λ* HLC ratio) and the patient remained asymptomatic, the dFLC levels increased to 1052 mg/L (*κ*/*λ* sFLC ratio: 0.008), indicating the reemergence of a *λ* FLC clone. Four months later, the patient progressed to symptomatic disease with severe renal impairment (creatinine 6.19 mg/dL; eGFR 7.03 mL/min/1.73 m^2^), anaemia (Hb 9.0 g/dL), and 70% clonal bone marrow plasma cells and the dFLC concentration had further increased to 9726 mg/L. However, the IgG*κ* monoclonal protein was no longer detectable by IFE and the IgG*κ*/IgG*λ* HLC ratio remained within the normal range, indicating that the biclonal MGUS had progressed to a *λ* light chain multiple myeloma.

### 2.2. Case 2

A 62-year-old woman presented with anaemia (haemoglobin; 9.2 g/dL) in March 2011 and was diagnosed with stage I oligosecretory IgG*κ* MM (SPE; 7.4 g/L, *κ* sFLC: 47.3 mg/L, *κ*/*λ* sFLC ratio 5.9). At this time, there was no uBJP detectable by urine protein electrophoresis (UPE) despite the bone marrow biopsy identifying 50% involvement by monoclonal *κ* restricted plasma cells. The IgG*κ*/IgG*λ* HLC ratio was abnormal (6.85). The patient was serially monitored with SPE, immunofixation electrophoresis (IFE), and sFLC throughout her disease course (Figure 1(b) and Table 1). She was initially treated with cyclophosphamide, thalidomide, and dexamethasone (CTD), although this treatment was halted after 3 cycles due to adverse side effects (increasing transaminases). The CTD treatment resulted in a similar decrease in the IgG*κ* monoclonal protein and the dFLC concentration (59% and 52%, resp.). Subsequently, she was treated with 8 cycles of bortezomib, doxorubicin, and dexamethasone (PAD). Although the IgG*κ* monoclonal protein concentration decreased by 73% to 2 g/L, the dFLC concentration initially decreased but ultimately increased to 274 mg/L. Following an autologous stem cell transplant (ASCT), the IgG*κ* monoclonal protein was still detectable by SPE (3.2 g/L) and the dFLC levels had decreased by 80% (dFLC 54.2 mg/L; *κ*/*λ* sFLC ratio 4.3). Eight months later, only trace quantities of the IgG*κ* monoclonal protein were detected by IFE (normalised IgG*κ*/IgG*λ* HLC ratio). However, the dFLC concentration increased to 326 mg/L (*κ*/*λ* sFLC ratio 26.5), indicating the outgrowth of a *κ* FLC producing clone. Six months later, she was diagnosed with progressive disease and severe renal impairment (eGFR MDRD; 8.73 mL/min/1.73 m^2^). At this time, the dFLC level had peaked at 2665 mg/L whilst the IgG*κ* monoclonal protein remained stable (trace quantities detected by IFE; normal IgG*κ*/IgG*λ* HLC ratio).

### 2.3. Case 3

A 70-year-old man, referred to our institute with rib fractures, was investigated and diagnosed with stage II IgA*κ* MM (SPE: 16 g/L, *κ* sFLCs: 3440 mg/L; *κ*/*λ* sFLC ratio 558) with 17% clonal bone marrow plasma cells. The patient's characteristics are described in Table 1. The patient was serially monitored with SPE, IFE, total IgA (due to the *β*-region migration of the monoclonal protein), and sFLC. Retrospective HLC analysis revealed an abnormal IgA*κ*/IgA*λ* HLC ratio at diagnosis (24.2) and this remained abnormal throughout the patient's disease course (Figure 1(c)). The patient was treated initially with vincristine, doxorubicin, and dexamethasone (VAD; 6 cycles) and achieved a PR. In March 2011, the patient was treated with 5 cycles of CTD and achieved a VGPR (SPE negative, IFE positive, normal total IgA concentration, dFLC 24 mg/L; *κ*/*λ* sFLC ratio 3.5). Due to the cardiac side effects of thalidomide, the patient was subsequently only treated with CD. Between March 2012 and July 2012, the patient was monitored by SPE only and during this time the IgA*κ* monoclonal protein remained stable (data not shown). In September 2012, progression of osteolysis was noted and whilst both SPE and total IgA measurements were uninformative, the dFLC levels increased from 24.5 mg/L to 821 mg/L (*κ*/*λ* sFLC ratio 103.5). The osteolytic lesions were irradiated with 2000 cGy/t, resulting in stable disease. However, in March 2013, the dFLC levels increased again (dFLC 938 mg/L, *κ*/*λ* sFLC ratio 157.9) indicating progressive disease and 1 month later, whilst the IgA*κ* monoclonal protein remained stable, progression of bone disease was identified. Treatment with 4 cycles of bortezomib, cyclophosphamide, and dexamethasone (VCD) was initiated and the patient achieved a VGPR (IgA*κ* trace by IFE, normal total IgA concentration and >90% reduction in dFLC). Treatment with VCD was interrupted due to severe neuropathy and Herpes viral infection. He then remained without treatment until clinical relapse in January 2014, characterised by renal impairment and hypercalcaemia. At this time, the IgA*κ* monoclonal protein remained stable (trace quantities by IFE, normal Total IgA concentration) but the dFLC had increased to 5404 mg/L, indicating relapse by the *κ* FLC producing clone.

## 3. Discussion

Disease progression from MGUS to MM is now thought to be the result of Darwinian-like evolution which leads to multiple clones being present at MM diagnosis, possibly producing different monoclonal proteins. The cases presented here indicate that disease progression and relapse may be associated with selective outgrowth of a FLC producing clone. The MGUS patient (case 1) had two separate clones present at diagnosis, one producing monoclonal IgG*κ* and another monoclonal *λ* FLC. Over time, the combined selective pressure applied by the bone marrow microenvironment and steroid treatment for PMR resulted in the *λ* FLC producing clone becoming dominant, leading to the development of LCMM. Similarly, the two MM cases highlight that the selective pressure of treatment (alongside the pressure applied by the microenvironment) resulted in the outgrowth of a more aggressive FLC producing clone.

In this study, all 3 cases relapsed with renal impairment. This result is in agreement with a previous study which reported renal impairment in 50% of patients with LCE escape [12]. As renal impairment is known to be associated with significant morbidity and mortality, it is important to identify LCE early. Interestingly, in this study we found that two of the three cases had an increase in their sFLC concentrations prior to clinical relapse (114 and 187 days), highlighting the clinical benefit of serially monitoring not only MM patients but also MGUS patients with sFLC to ensure early identification of disease relapse/progression and help prevent the development of irreversible end-organ damage. Supporting this, the study of Brioli et al. reported that patients relapsing with sFLC involvement (either LCE or alongside an increase in the intact immunoglobulin) had a significantly shorter overall survival compared to those patients relapsing without a sFLC component [10].

It is noteworthy that, in case 1 presented here, one year after steroidal treatment for PMR, the patient's sFLC ratio was highly abnormal (0.008; involved/uninvolved ratio = 125) although they remained asymptomatic. The recently updated IMWG criteria for the definition of multiple myeloma no longer require a patient to display symptoms of end-organ damage in the presence of one or more biomarkers of malignancy (plus at least 10% clonal bone marrow plasma cells/biopsy proven bony or extramedullary plasmacytoma), one of which being a sFLC involved/uninvolved ratio of 100 [13]. Therefore, had these updated criteria for myeloma definition been in effect at the time the patient described in case 1 was monitored, upon observation that the sFLC involved/uninvolved ratio had increased to 125, it is tempting to speculate that this may well have prompted further investigation to look for evidence of progression to myeloma. Importantly, this was some 4 months before the patient developed symptomatic disease, and if progression to asymptomatic myeloma had been confirmed at this time, this may have allowed earlier therapeutic intervention to help prevent the severe renal impairment that the patient ultimately acquired.

The IMWG guidelines recommend that, for patients with oligosecretory MM (<10 g/L or <200 mg/24 h M-protein), response should be assessed by sFLC, if the involved FLC (iFLC) is ≥100 mg/L [14]. In case 2 (SPE 7.4 g/L M-protein, uBJP negative), the iFLC concentration was 47.3 mg/L and therefore the IMWG guidelines recommend monitoring such patients with BMPCs. However, this case highlights that sFLC analysis is still a valuable monitoring tool even if the iFLC is below 100 mg/L as the increase in the dFLC identified disease progression 6 months prior to development of renal impairment. In addition, this case highlights that HLC analysis could be used alongside sFLC to monitor the M-Ig producing clone in oligosecretory disease. This is supported by a number of other studies which have shown that HLC assays can be used to accurately monitor oligosecretory patients [15–17], especially those with an iFLC < 100 mg/L [17].

IgA monoclonal proteins that migrate within the *β* region by SPE can be difficult to identify and/or accurately quantify due to comigration with other serum proteins (such as transferrin and complement proteins). Although the use of nephelometric/turbidimetric immunoglobulin quantification alongside electrophoresis is recommended by the IMWG guidelines, when total IgA measurements fall within the normal range, it is not clear if a monoclonal protein is still present [18]. This is illustrated by case 3 presented here, as the total IgA measurements were borderline normal throughout the patient's disease course and therefore uninformative. However, the HLC ratio remained abnormal throughout the patient's disease course indicating the presence of a monoclonal IgA*κ* producing clone that remained stable and was not responsive to any of the therapies. The HLC ratio has been shown previously to be an accurate measurement of clonality and useful in the monitoring of MM patients [16, 19]. Furthermore, a recent study from Katzmann and colleagues concluded that IgA HLC immunoassays can be used instead of SPE, IFE, and total IgA quantification for monitoring *β*-region migrating IgA monoclonal proteins, as was the situation in case 3 presented here [20].

In conclusion, the cases presented here highlight the importance of being able to monitor clonal evolution over the course of the disease in multiple myeloma, particularly as disease progression in the form of LCE can result in rapid and irreversible end-organ damage that may be preventable if the disease progression can be detected early. To this end, the combined use of more sensitive monitoring tools, such as using sFLC and HLC assays together, may fulfill this requirement and permit closer monitoring of multiple myeloma patients.

## Figures and Tables

**Table 1:** Patient characteristics for three cases of progression to LCMM by light chain escape.

	Case 1	Case 2	Case 3
Age (years)	71	62	70
Gender	F	F	M
MGUS/MM type	IgG*κ* + *λ* FLC	IgG*κ*	IgA*κ*
M-protein by SPE at diagnosis (g/L)	3.9	7.4	16
HLC ratio at diagnosis	*4.8*	*6.9*	*24.2*
iFLC [*κ*/*λ* ratio] at diagnosis (mg/L)	*316 *[*0.07*]	*47.3 *[*5.9*]	*3440 *[*558*]
uBJP at diagnosis	+	−	+
ISS stage	N/A	I	II
Symptoms at relapse	Renal impairment	Renal impairment	Renal impairment
dFLC concentration at relapse (mg/L)	9726	2665	5404
Time prior to clinical relapse that increases in FLC detected (days)	114	187	0
Maximum response	N/A	PR	VGPR
Treatment	Methylprednisolone	CTD, PAD, and ASCT	VAD, CTD, CD, IR, and VCD
Follow-up (days)	1310	1019	1570

LCMM: light chain multiple myeloma, F: female, M: male, N/A not applicable, MGUS: monoclonal gammopathy of undetermined significance, MM: multiple myeloma, SPE: serum protein electrophoresis, HLC: heavy/light chain assay (Hevylite), iFLC: involved free light chain concentration (Freelite), uBJP: urinary Bence Jones protein, ISS: international staging system, dFLC: difference in concentration between involved and uninvolved free light chain measurement (Freelite), FLC: free light chains, PR: partial response, VGPR: very good partial response, ND: not determined, CTD: cyclophosphamide, thalidomide, and dexamethasone, PAD: bortezomib, doxorubicin, and dexamethasone, ASCT: autologous stem cell transplant, VAD: vincristine, doxorubicin, and dexamethasone, VCD: bortezomib, cyclophosphamide, and dexamethasone, CD: cyclophosphamide and dexamethasone, and IR: ionising radiation.
